# A survey of HIV infection and related high-risk factors among men who have sex with men in Suzhou, Jiangsu, China^☆^

**DOI:** 10.1016/S1674-8301(11)60002-X

**Published:** 2011-01

**Authors:** Hongling Bai, Xiping Huan, Weiming Tang, Xin Chen, Hongjing Yan, Xiaoyan Liu, Haitao Yang, Zhihang Peng, Xiuping Zhao, Rongbin Yu, Hao Yu, Feng Chen

**Affiliations:** a Department of Epidemiology and Biostatistics, School of Public Health, Nanjing Medical University, Nanjing, Jiangsu 210029, China;; b Center for Disease Control and Prevention of Jiangsu Province, Nanjing, Jiangsu 210009, China;; c Suzhou Center for Disease Prevention and Control, Suzhou, Jiangsu 215004, China.

**Keywords:** men who have sex with men, knowledge, behavior, HIV infection, risk factor

## Abstract

A cross-sectional study using the snowball sampling method was conducted in May 2008 to investigate human immunodeficiency virus (HIV) infection status and related high risk factors among men who have sex with men (MSM) in Suzhou city of Jiangsu province. The researchers carried out a face-to-face questionnaire interview among MSM, and collected their blood samples to test for HIV and other sexually transmitted diseases (STDs). Among the 280 respondents, 91.1% had homosexual acts in the past 6 months and 87.5% had multiple homosexual partners; 46.4% had heterosexual sex in the past 6 months and 33.1% had multiple heterosexual partners. The rate of continued condom use was 44.3% in homosexual sex in the past 6 months, while the rate in heterosexual sex was 33.9%. Laboratory test results showed that the prevalences of HIV and syphilis were 7.1% (20/280) and 15.0% (42/280), respectively, but no HCV-positive person was found. In the multivariate logistic regression model, subjects with a monthly income of more than RMB ¥ 1,000 (*OR*=4.83,95% *CI*=1.44-16.22), subjects who often went to bars for sexual partners (*OR*=2.25, 95%*CI*=1.21-4.20), and subjects who had more than one sexual partner in the past 6 months (*OR*=0.49, 95%*CI*=0.25-0.97) and had sex with fixed sexual partners in the past 6 months (*OR*=0.42, 95%*CI*=0.25-0.75) were significantly associated with the rate of continued condom use in homosexual sex in the past 6 months. Unprotected sex and multiple sexual partners were more common among MSM in Suzhou city; furthermore, the prevalences of HIV infection and syphilis were relatively high. HIV preventive measures should be designed to address these risk factors and control the spread of HIV among MSM.

## INTRODUCTION

Men who have sex with men (MSM) are known to belong to one of the three high-risk groups for infection with human immunodeficiency virus (HIV)[1]–[3]. The morbidity of acquired immune deficiency syndrome (AIDS) and the proportion of HIV infection in the population have been increasing year by year[4]–[7] and MSM have become an increasingly critical population in the HIV epidemic in China. Growing public acceptance of homosexuality in China allows MSM to connect closely with one other and meet a great number of sex partners through the Internet and gay bars that have been increasing in number in China, clubs and other venues, which could be the main cause of the rapid spread of HIV across the population. In contrast to countries where the majority of MSM do not have sex with women, many MSM in China are married or have multiple female partners, and it increases the possibility for a higher rate of heterosexual transmission of HIV. Domestic and overseas researches revealed that unprotected sex, multiple sexual partners and other high-risk behaviors are more common among MSM and lead to a high prevalence of HIV/AIDs in this special population[4]–[10]. Although Jiangsu province belongs to low epidemic area of AIDS, recent studies indicate that this area has a sustained growth in HIV/AIDS infection, especially in MSM[11]. Suzhou is a large city located in the middle of Jiangsu province. The downtown traffic is convenient and there are many taprooms, bathrooms, parks and so on, parts of which have been the gathering places of MSM[12]. It was estimated that about 2,000 to 3,000 MSM gathered in fixed sites according to the information provided by the site owner not long before. This cross-sectional survey aimed to know about high-risk behaviors, and the status of HIV/AIDS and other sexually transmitted diseases (STDs) in MSM within the jurisdiction of Suzhou by qualitative questionnaire and serological surveys among the MSM population. At the same time, we sought to analyze the determining factors in order to provide a scientific basis to draft HIV/AIDS prevention strategies aimed at the MSM population.

## MATERIALS AND METHODS

### Study subjects

A cross-sectional study using the snowball sampling method and internet broadcast was conducted in May 2008 in Suzhou to cramp out MSM in accordance with the following standards: subjects who had settled in Suzhou for more than three months; subjects who had oral or anal homosexual behavior in the past year; subjects who aged 18 years and above. The study was approved by the Ethics Cominittee at each author's affiliated institutions and all subjects were enrolled with an informed consent.

### Laboratory tests

Five mL of venous blood sample from the subjects were collected to test for HIV, HCV-antibody (Ab), *Treponema Palladium* antibody and other STDs.

HIV-Ab was prescreened by enzyme-linked immune sorbent test method (ELISA) and confirmed by Western blotting assay. The syphilis antibody was prescreened by rapid plasma response meat/toluene detection method (RPR/TRUST) and confirmed by the *Treponema Palladium* gelatin agglutinate experiment method (TPPA). HCV antibody was prescreened by ELISA, and confirmed by the recombinant immune imprinting screening method (RIBA).

### Statistical analysis

Data was doubly entered into a computerized database using EpiData 3.1 software. Statistical analysis was performed using Stata 10.0. Descriptive analysis was conducted on demographic and behavioral characteristics and syphilis infection. Variables (*P* < 0.10) significant in the bivariate analysis were considered for inclusion in multivariate logistic regression models.

## RESULTS

### Basic characters

Totally, 280 MSM met the inclusion standard and were recruited in this survey, and all finished the questionnair. The average age was 28.1±8.8 years. One huandred and eighty-five (66.1%) were unmarried; 81 (28.9%) were married; 5 (1.8%) were unmarried couples; 9 (3.2%) were divorced or widowed. Sixty-eight (24.3%) were natives of Suzhou; 72 (25.7%) were from other districts of Jiangsu province, and 140 (50.0%) were from other provinces. Two hundres and seventy-eight (99.3%) were Han nationality. Four (1.4%) finished primary school; 56 (20.0%) finished junior high school; 114 (40.7%) received senior high school or technical secondary school; 106 (37.9%) received junior college or above education. Fifteen (5.4%) reported no income; 5 (1.8%) had an income of ¥1000 per month or below, 93 (33.2%) had ¥1001-2000 per month, 80 (28.6%) had ¥2001-3000 per month, 40 (14.3%) had 3001-4000 per month, 47 (16.8%) had ¥4000 or above per month.

### Knowledge of and attitude towards HIV/AIDS

The respondents had a relatively good knowledge of HIV/AIDS with an accuracy rate of 87.5%-98.6%, but for the question “whether mosquito will spread the AIDS virus”, it had a lower accuracy rate of 56.8%. As for HIV/AIDS attitude, among 280 MSM, 243 people (86.8%) thought that HIV/AIDS is much more horrible than other chronic diseases, and 232 (82.9%) considered using condoms to prevent HIV infection. Twenty-two (7.9%) still chose anal sex without condom though they knew that there was a high infection rate of HIV/AIDS. One hundred and fifty (53.6%) thought that they had no chance of being infected by HIV in the next year.

### Sexual behavior characters

The respondents had their first sexual behavior at an average age of 21.5±6.0 years. One hundred and twenty-four (44.3%) of them had their first sex act with man at an average age of 23.8±7.6 years. The respondents with homosexual orientation were 115 (41.1%), with heterosexual orientation were 12 (4.3%), and with bisexual orientation were 144 (51.4%), and 9 people (3.2%) had undetermined sexual orientation.

### Homosexual acts

Among 280 MSM, 255 people (91.1%) responded that they had anal sex with male partners in the recent six months with an average of 7.5 sexual partners. Two hundred and thirty-three people (87.5%) had 2 or more sexual partners. Among the respondents, 87 (34.1%) chose a general role as “inserter”, 39 (15.3%) chose “receiver”, and 129 (50.6%) chose both. For sex acts in the recent six months, 17 persons (6.7%) never used condom; 125 persons (49.0%) used condom sometimes; only 113 persons (44.3%) used condom regularly. For the last sex behavior, 201 persons (78.8%) used condom. One hundred and forty-nine (58.4%) responded that they had homosexual act with stable sexual partners in the recent six months, of which 60 (40.3%) had anal sex with fixed partners by regular condom use in the recent six months. Two hundred subjects (78.4%) had unfixed sexual partners, of which 104 (52.0%) had anal sex with unfixed partners by regular condom use in the recent six months.

### Commercial sexual acts

Among 255 respondents, 32 (12.5%) had commercial sexual acts in the recent six months with an average of 31.0 sexual acts. Fifteen respondents (46.9%) had 2 or more than 2 commercial sexual partners, 28 (87.5%) used condom in the last commercial sexual act, and 24 (75.0%) had commercial sexual acts with condom every time in the recent six months. At the same time, 65 persons (25.5%) offered commercial sexual acts in the recent six months with an average number of 48.3, and 58 (89.2%) offered commercial sexual acts to 2 or more than 2 sexual partners. Sixty (92.3%) persons used condom in the last commercial sexual act, and 52 (80.0%) had commercial sexual acts with condom regularly in the recent six months.

### Heterosexual acts

Among 280 MSM, 130 (48.7%) responded that they had heterosexual acts with an average of 1.7 and 43 (33.1%) have 2 or more than 2 heterosexual partners. Sixty-three (48.5%) used condom during the last heterosexual act. Forty-seven (36.2%) never used condom in the recent six months during the last heterosexual acts, and 39 (30.0%) sometimes used, and only 44 (33.9%) used condom regularly.

### Situations of STDs/AIDS infection

Blood samples from the subjects were collected for laboratory testing. It was confirmed that 20 persons were HIV positive with a positive rate of 7.1% (95%*CI*=4.41-10.82), 42 persons were positive for syphilis with a positive rate of 15.0% (95%*CI*=11.02-19.73), and no persons were found positive for HCV in the screening.

### Analysis of MSM sociodemographic characters, sexual behaviors and sex condom use rate in the past 6 months by single factor χ^2^

Single factor χ^2^ analysis results showed that the variables that had statistically significant relationship with the rate of condom use in homosexual acts in the past six months were as follows: age, civil state, census register, often going to the bars for sexual partners, age of the first insert sexual behavior, the number of homosexual partners and having sex with fixed partners in the past 6 months shown in ***Table 1*** and ***Table 2*** (*P* < 0.05).

**Table 1:** Socioenonomic and demographic characteristics and condom use of the study participants in the previous six months

Factors	Number	Consistent condom use [*n*(%)]	*χ*^2^	*OR*	95% *CI*	*P*
Age (years)						
≤23	100	50 (50.0)	6.01	1	0.32-0.91	0.0142
>23	180	63 (35.0)		0.54		
Civil state						
Unmarried/divorced or widowed	194	88 (45.4)	6.57	1	0.17-0.88	0.0104
Married/cohabitant	86	25 (29.1)		0.49		
Census register						
Not native	212	94 (44.3)	5.75	1	0.25-0.91	0.0165
Suzhou native	68	19 (27.9)		0.49		
Educational level						
Under the high school	60	22 (36.7)	0.43	1	0.65-2.32	0.5110
High school and above	220	91 (41.4)		1.22		
Monthly income						
≤¥1,000 (RMB)	20	4 (20.0)	3.71	1	0.90-12.16	0.0541
>¥1,000 (RMB)	260	109 (41.9)		2.89		

CI: confidence interval; OR: odds ratio.

**Table 2:** Single factor analysis of sexual behaviors and consistent condom use in MSM by in the previous six months

Factors	Number	Consistent condom use [*n*(%)]	*χ*^2^	*OR*	95% *CI*	*P*
Sexual orientation						
Homosexual	165	73 (44.2)		1		
Heterosexual	115	40 (34.8)	2.52	0.67	0.40-1.13	0.1124
Role in recent six months						
Both	129	55 (42.6)		1		
Inserting	87	43 (49.4)	0.97	1.31	0.73-2.35	0.3256
Receptive	39	15 (38.5)	0.21	0.84	0.37-1.85	0.6431
Often go to the bars for sexual partners						
No	206	75 (36.4)		1		
Yes	74	38 (51.4)	5.05	1.84	1.04-3.27	0.0246
Often go to the bathrooms for sexual partners						
No	232	98 (42.2)		1		
Yes	48	15 (31.3)	2.00	0.62	0.30-1.25	0.1577
Often go to the parks for sexual partners						
No	275	112 (40.7)		1		
Yes	5	1 (20.0)	0.88	0.36	0.01-3.75	0.3492
Often look for sexual partners through the Internet						
No	147	64 (43.5)		1		
Yes	133	49 (36.8)	1.30	0.76	0.45-1.26	0.2541
The age of the first insert sexual behavior (years)						
≤21	162	75 (46.3)		1		
>21	118	38 (32.2)	5.63	0.55	0.33-0.93	0.0176
The age of first homosexual behavior (years)						
≤21	129	56 (43.4)		1		
>21	151	57 (37.7)	0.93	0.79	0.48-1.31	0.3357
First sexual partner						
Female	156	66 (42.3)		1		
Male	124	47 (37.9)	0.56	0.83	0.50-1.39	0.4556
Number of homosexual partners in the recent 6 months						
1	53	27 (50.9)		1		
≥2	202	86 (42.3)	5.88	0.46	0.24-0.91	0.0153
Have sex with fixed sexual partner						
No	106	57 (53.8)		1		
Yes	149	56 (37.6)	6.58	0.52	0.30-0.89	0.0103
Have sex with unfixed sexual partners						
No	55	28 (50.9)		1		
Yes	200	85 (42.5)	1.24	0.71	0.37-1.36	0.2662
Have commercial sexual acts						
No	174	68 (39.1)		1		
Yes	81	45 (42.5)	0.31	1.15	0.68-1.93	0.5769
Have heterosexual acts						
No	150	61 (40.7)		1		
Yes	130	52 (40.0)	0.01	0.97	0.59-1.61	0.9097

CI: confidence interval; OR: odds ratio.

### Multiple factor logistic regression analysis of condom use in MSM during homosexual acts in the recent six months

Those variables that were significant (*P* < 0.10) in bivariate analysis were considered for inclusion in a multiple logistic regression model; as a result, variables that entered the multiple factors logistic regression model were as follows: monthly income more than RMB¥ 1,000 (*OR*=4.83,95%*CI*=1.44-16.22), often going to the bars for sex (*OR*=2.25, 95%*CI*=1.21-4.20), having more than one sexual partner in the past 6 months (*OR*=0.49, 95%*CI*=0.25-0.97) and having sex with fixed sexual partner in the past 6 months (*OR*=0.42,95%*CI*=0.25-0.75) (***Table 3***).

**Table 3:** Multiple factors logistic regression analysis of MSM with condom usage for homosexual behavior in the previous six months

Factor	Regression coefficient	Standard error	*OR* (95% *CI)*	*P*
Monthly income> ¥1,000 (RMB)				
yes=l, no=0	1.5757	0.6177	4.83 (1.44-16.22)	0.0180
Often go to the bars for sexual partners				
yes=l, no=0	0.8111	0.3179	2.25 (1.21- 4.20)	0.0110
Have more than one sexual partners in the recent six months				
yes=l, no=0	-0.7069	0.3469	0.49 (0.25- 0.97)	0.0420
Have sex with fixed sexual partner in the recent six months				
yes=l, no=0	-0.8585	0.2906	0.42 (0.24- 0.75)	0.0030

CI: confidence interval; OR: odds ratio.

### Single factor analysis of sexual features of MSM, infection of syphilis and social demographic characters with HIV infection

The analysis results showed that statistically significant variables of HIV infection included the number of homosexual partners and having unfixed homosexual partners within six months shown in ***Table 4*** and ***Table 5*** (*P* < 0.05).

**Table 4:** Univariate analysis of the relationship among social demographic characteristics, syphilis and HIV infections in MSM

Factors	Number	HIV positive [*n*(%)]	*χ*^2^	*OR*	95% *CI*	*P*
Age (years)						
≤23	100	6 (6.0)		1		
>23	180	14 (7.8)	0.31	1.32	0.46-4.33	0.5799
Civil state						
Unmarried/divorced or widowed	194	12 (6.2)		1		
Married/cohabitate	86	8 (9.3)	0.87	1.56	0.53-4.32	0.3502
Census register						
Not native	212	14 (6.6)		1		
Suzhou native	68	6 (8.8)	0.38	1.37	0.41-3.99	0.5363
Educational level						
Below high school	60	3 (5.0)		1		
High school and above	220	17 (7.7)	0.53	1.59	0.44-8.75	0.4672
Monthly income						
≤ ¥1,000 (RMB)	20	2 (10.0)		1		
> ¥1,000 (RMB)	260	18 (6.9)	0.27	0.67	0.14-6.41	0.6066
Infection of syphilis						
No	238	13 (5.5)		1		
Yes	42	7 (16.7)	6.76	3.46	1.08-10.07	0.0093

CI: confidence interval; OR: odds ratio.

**Table 5:** Univariate analysis of the characteristics of sex behaviors and HIV Infections in MSM

Factors	Number	HIV positive [*n*(%)]	*χ*^2^	OR	95% CI	*P*
Sexual orientation						
Homosexual	165	10 (6.1)		1		
Heterosexual	115	10 (8.7)	0.71	1.48	0.53-4.10	0.3996
Role in the recent six month						
Both	129	12 (9.3)		1		
Inserting	87	3 (3.4)	2.76	0.35	0.06-1.35	0.0969
Receptive	39	5 (12.8)	0.41	1.43	0.37-4.75	0.5232
Often go to the bar for sexual partners						
No	206	13 (6.3)		1		
Yes	74	7 (9.5)	0.81	1.55	0.50-4.39	0.3670
Often go to the bathroom for sexual partners						
No	232	15 (6.5)		1		
Yes	48	5 (10.4)	0.94	1.68	0.45-5.20	0.3333
Often go to the park for sexual partners						
No	275	20 (7.3)		/		
Yes	5	0 (0)	0.39	/	/	1.0000
Often look for sexual partners through the Internet						
No	147	12 (8.2)		1		
Yes	133	8 (6.0)	0.49	0.72	0.25-1.99	0.4858
Age of the first insert sexual behavior (years)						
≤21	162	12 (7.4)		1		
>21	118	8 (6.8)	0.04	0.91	0.31-2.51	0.8404
The age of first homosexual behavior (years)						
≤21	129	12 (9.3)		1		
>21	151	8 (5.3)	1.68	0.55	0.19-1.51	0.1947
First sexual partner						
Female	156	9 (5.8)		1		
Male	124	11 (8.9)	1.00	1.59	0.58-4.49	0.3168
Homosexual partners in the recent 6 month						
1	53	0 (0)		/		
≥2	202	19 (9.4)	5.39	/	/	0.0160
Have sex with fixed sexual partner						
No	106	8 (7.5)		1		
Yes	149	11 (7.4)	0.00	0.98	0.34-2.91	0.9607
Have sex with unfixed sexual partner						
No	55	0 (0)		/		
Yes	200	19 (9.5)	5.65	/	/	0.0170
Have commercial sexual acts						
No	174	11 (6.3)		1		
Yes	81	8 (9.9)	1.01	1.62	0.54-4.64	0.3142
Have heterosexual act						
No	150	11 (7.3)		1		
Yes	130	9 (6.9)	0.02	0.94	0.33-2.59	0.8942

### Analysis of HIV infection by multiple factors logistic regression

The factor variables whose result was *P* < 0.1 in the above single factor χ^2^ analysis were analysed by multiple factors logistic regression model; as a result, no variable entered the multiple factors logistic regression model.

## DISCUSSION

This survey of MSM in Suzhou showed that the average age of the MSM was 28.1 years, and the average age at first sex with man was 23.8 years, which was older than that in other researches[13]–[15]. There may be a chance for younger MSM to flow and gather to big cities like Nanjing. The MSM in Suzhou were born early on average so that their first homosexual behavior occured later, which is consistent with the result of Li *et al*[16]. On the other hand, the percentage of first sexual behavior with man in this research was 44.3%, which was lower than Li *et al.* result. The reason might be that conservative sex concept in Suzhou led MSM to develope a sense of their sex orientation late.

The awareness of knowledge on HIV/STDs by MSM in Suzhou in this research was 87.5%-98.6%, which is similar to that of other regions[17]. But they still do not master the prevention knowledge very well and in the aspect of attitude to HIV/AIDS, 7.9% persons responded that they would not use condom for anal sex though they knew that there would be a high chance of HIV infection. Fifty-three point six percent of them believed that they would be infected with HIV in the following year. It is pointed out that we should provide a comprehensive knowledge of HIV/AIDS including the process and the result of the disease when we broadcast, and consequently educate the public especially the mode of transmission of the disease.

In the aspects of HIV/AIDS related behaviors, firstly, the rate of condom use within Suzhou MSM was apparently higher than that of other cities[18],[19], and there was no association between knowledge and action. It indicated that earlier intervention on behavior should be efficient. We found in this survey that the rate of consistent condom use by MSM was 78.4% when they had anal sex with fixed partner. Logistic regression analysis of multiple factors showed that having sex with fixed sex partner in the recent six months and the rate of consistent condom use were negatively correlated, which is in accordance with other surveys in China[20]. It may be that MSM tend to trust their sex partners or they do not use condom in fear of hurting each other. In this survey, the average number of sex partners reached up to 7.9, and 87.5% of them had more than one partner, which was higher than the result of research conducted in Shenzhen by Cai *et al*[21]. At the same time, 46.9% and 89.2% of MSM had more than one sexual partner in buying and selling sex, respectively, which was relatively high. It indicates that it is common for MSM in Suzhou to have more than one partner, and it implied that we should enhance education to guide and encourage MSM to keep fixed sexual partner.

Early researches showed that subgroups of MSM looking for sexual partners through the Internet had more sexual partners and more unprotected homosexual behaviors[22],[23]. However, in this study, 36.8% of MSM looking for sexual partners through the Internet, and only 6% were HIV positive. The Internet is a venue for looking for sexual partners as well as a tool to broadcast prevention knowledge of HIV/AIDS, which implied that we should better utilize the cheap but efficient carrier to educate the MSM.

Commercial sexual behavior and heterosexual behavior group are two subgroups of MSM and they are more likely to spread AIDS because most of them had multiple sex partners and are engaged in unprotected insert sexual behaviors[24]. Forty-one percent of them had sex trade in the recent six months in this survey, which was higher than that reported in the research (22.2%) in Shenzhen by Cai *et al.*[21] and research (10.6%) in Beijing by Li *et al*[16]. It also showed that the rate of condom use in MSM who had commercial sexual acts was higher than others, indicating that this group pay more attention to their health, which benefits the prevention of AIDS. In the aspect of heterosexual behavior, 46.6% of them had heterosexual acts in the recent six months, which is higher than that of other cities[25],[26]. The rate of marriage was 28.9%, which is relatively low. The rate of consistent condom use is 33.9%, which is similar to that of other research results. It indicated that heterosexual behavior in MSM in Suzhou is common and mostly unprotective[27],[28], so it probably may be a bridge to allow HIV to spread to the general population. These findings highlighted a great need for behavioral interventions, focusing on the potential bridging subgroup to prevent the fueling of a heterosexual epidemic spread of HIV.

For laboratory tests, the HIV infection rate of MSM in Suzhou was 7.1%, which is higher than 5.8% reported by the 2006-2007 research in Jiangsu, and the infection rate of syphilis is 15.0%, which is lower than the latter (27.7%). This suggestes that though Jiangsu province is an area with low HIV infection rate, research and intervention measures aimed at MSM still need to be carried out early. Early effective prevention and intervention play an important role in reducing the infection rate of syphilis. While infection of HIV has many complex related factors, and it will be a long and systemic project to prevent and control, it can be predicted that infection of AIDS in the target population will increase in the short run. In this survey, multiple factors logistic regression analysis of HIV infection showed that no factor is related with HIV infection, but infection of syphilis and HIV are positively correlated. It shows that infections of syphilis and other STDs make MSM more susceptible to HIV. In addition, many researches have proved the possibility that HCV can be spread through sex and have a high infection rate especially among the high risk population. Due to the quality of HCV detection, we did not find any HCV positive subject in our research.

There are also some drawbacks in our research. For example, we adopt a kind of nonrandom and improbability snowball sampling method, which impairs the validity of the research. In fact, we can use respondent driven sampling (RDS) method, which is now widely used in MSM and other populations that are difficult to keep track of. With this, we can enlarge the number of samples to do more profound research that can cover every sub-MSM population so that we can describe the AIDS related behavior characteristics and prevalent condition of MSM in the whole Suzhou city, and even the whole Jiangsu province. Further researches on the related hazards are needed to offer more evidence for HIV/AIDS prevention and control.
